# From AI-based image analysis to surgical decision support in prostate cancer: interdisciplinary application of the international radiomics platform

**DOI:** 10.3389/fonc.2026.1816463

**Published:** 2026-05-29

**Authors:** Fabian Tollens, Niklas Westhoff, Jan Moltz, Tim Hartenstein, Anne Sophie Michel, Mahnoosh Naeimi, Johannes Ludwig, Peter Kohlmann, Judith Herrmann, Konstantin Nikolaou, Stefan O. Schoenberg, Dominik Nörenberg

**Affiliations:** 1 Department of Radiology and Nuclear Medicine, University Medical Center Mannheim, Heidelberg University, Mannheim, Germany; 2 Department of Urology and Urosurgery, University Medical Center Mannheim, Heidelberg University, Mannheim, Germany; 3 Fraunhofer Institute for Digital Medicine MEVIS, Bremen, Germany; 4 Department of Radiology, University Hospital of Erlangen, Erlangen, Germany; 5 Department of Diagnostic and Interventional Radiology, Medical Faculty and University Hospital, Eberhard Karls University, Tübingen, Germany

**Keywords:** machine learning, multiparametric MRI, outcome prediction, PI-RADS, prostate cancer, radiomics

## Abstract

**Objectives:**

Diagnosis and surgical therapy planning for prostate cancer patients are often hindered by fragmented workflows and a lack of integrated, data-driven analysis - barriers that specifically impede the effective deployment of machine learning (ML)-based risk stratification and clinical decision support. The aim of this exploratory study was to clinically implement and prospectively validate a platform-based multimodal data analysis pipeline within a radiological-urological collaboration.

**Materials and methods:**

In this single center analysis, a total of 249 patients (176 retrospectively, 73 prospectively) undergoing radical prostatectomy were included. Multimodal datasets, including preoperative multiparametric MRI, clinical, laboratory, and pathological data, were harmonized and imported into the International Radiomics Platform (IRP). Radiomics features were extracted and machine learning models were constructed to predict extracapsular extension (ECE), nerve-sparing approach decision, and positive surgical margin (PSM) risk. Results are reported as area under the curve (AUC) values including 95% confidence intervals.

**Results:**

A cloud-based software prototype was implemented based on the IRP, integrating prediction models for clinical decision support. Using a step-wise modeling approach, prediction of extracapsular extension (ECE) improved substantially when imaging-derived parameters (e.g. PI-RADS scores, tumor-capsule contact length) were added to conventional clinical parameters (AUC 0.90, 95% CI: 0.86–0.94 vs. 0.71, 95% CI: 0.63–0.77). In contrast, the addition of imaging-derived features provided no meaningful incremental value for predicting positive surgical margins (PSM; AUC 0.60, 95% CI: 0.52–0.68) or nerve-sparing approach decisions (AUC 0.79, 95% CI: 0.73–0.83), which were also unchanged by the further inclusion of quantitative radiomics features. Performance was consistent across internal cross-validation and prospective external validation.

**Conclusion:**

This exploratory study demonstrates the feasibility of a platform-based, multimodal data analysis workflow for prostate cancer surgical planning. Integration of imaging-derived parameters meaningfully enhanced ECE prediction, while radiomics offered no additional benefit beyond standard imaging. These findings highlight both the potential and current limitations of AI-driven workflow integration in routine clinical practice.

## 1 Introduction

Prostate cancer is the most common non-cutaneous malignant tumor in men and the second most cancer-related cause of death in men worldwide (1). Multiparametric MRI (mpMRI) of the prostate has become an integral part of the diagnostic pathway, as it facilitates the detection of suspicious lesions for targeted biopsy and supports individualized surgical planning (2, 3). Accurate evaluation of extracapsular extension (ECE) and involvement of neurovascular bundles (NVB) is critical for optimizing operative strategies and risk stratification (4, 5). Positive surgical margins (PSM) are a well-established prognostic marker, closely linked not only to increased local recurrence and metastatic risk but also to reduced cancer-specific and overall survival (6, 7).

Despite technological advances, treatment planning for prostate cancer patients remains hindered by workflow fragmentation, absence of data integration, and a lack of standardized analytic pipelines (8). These barriers limit the effective, machine learning (ML)-based assessment and risk stratification required for precision treatment. A unified software environment – encompassing automated image reading, quantitative image analysis, structured reporting, and comprehensive integration of radiological, histopathological, and clinical data – has the potential to accelerate reporting workflows, improve multidisciplinary communication, and facilitate more reliable and actionable, data-driven clinical decision support (8).

Encouragingly, the European Society of Urogenital Radiology (ESUR) supports the development and validation of AI and radiomics solutions for mpMRI-based prostate cancer management, with a focus on workflow integration and patient-specific risk prediction to enable personalized diagnoses based on multivariate risk models (9).

The aim of this exploratory, proof-of-concept study was to evaluate the utility of a harmonized, cloud-based data science platform – the International Radiomics Platform (IRP) (10) – in an integrated diagnostic workflow of patients undergoing prostate cancer evaluation. The IRP permits standardized, reproducible analysis of imaging and clinical data, directly addressing existing data silos and workflow inefficiencies that currently impede ML deployment.

In this work, the IRP was specifically extended to support outcome prediction for surgical planning in prostate cancer, leveraging multimodal features and ML algorithms to predict the likelihood of PSM and ECE preoperatively. We prospectively implemented and validated our workflow along routine patient care, highlighting translational pathways for real-world surgical planning and precision oncology.

## 2 Material and methods

This study was conducted in accordance with the Declaration of Helsinki and approved by the local Ethics Committee (2020-581N). Written informed consent was obtained from all prospectively enrolled patients.

### 2.1 Patients

This single center prospective study recruited patients from University Medical Center Mannheim (UMM), Germany. Inclusion criteria comprised biopsy-proven prostate cancer (PCa) in men scheduled for radical prostatectomy, with pre-biopsy multiparametric MRI showing at least one suspicious lesion (PI-RADS ≥ 3).

A total of 249 patients were included, with 176 retrospectively identified who underwent radical prostatectomy from 2015 to 2019, and 73 men prospectively enrolled from 2021 to 2022. To comply with ethical requirements not to include patients before the treatment decision, patients were prospectively included right after radical prostatectomy to eliminate any possible influence of experimental prediction models on treatment choice.

### 2.2 Data integration and data security

**Figure 1:**
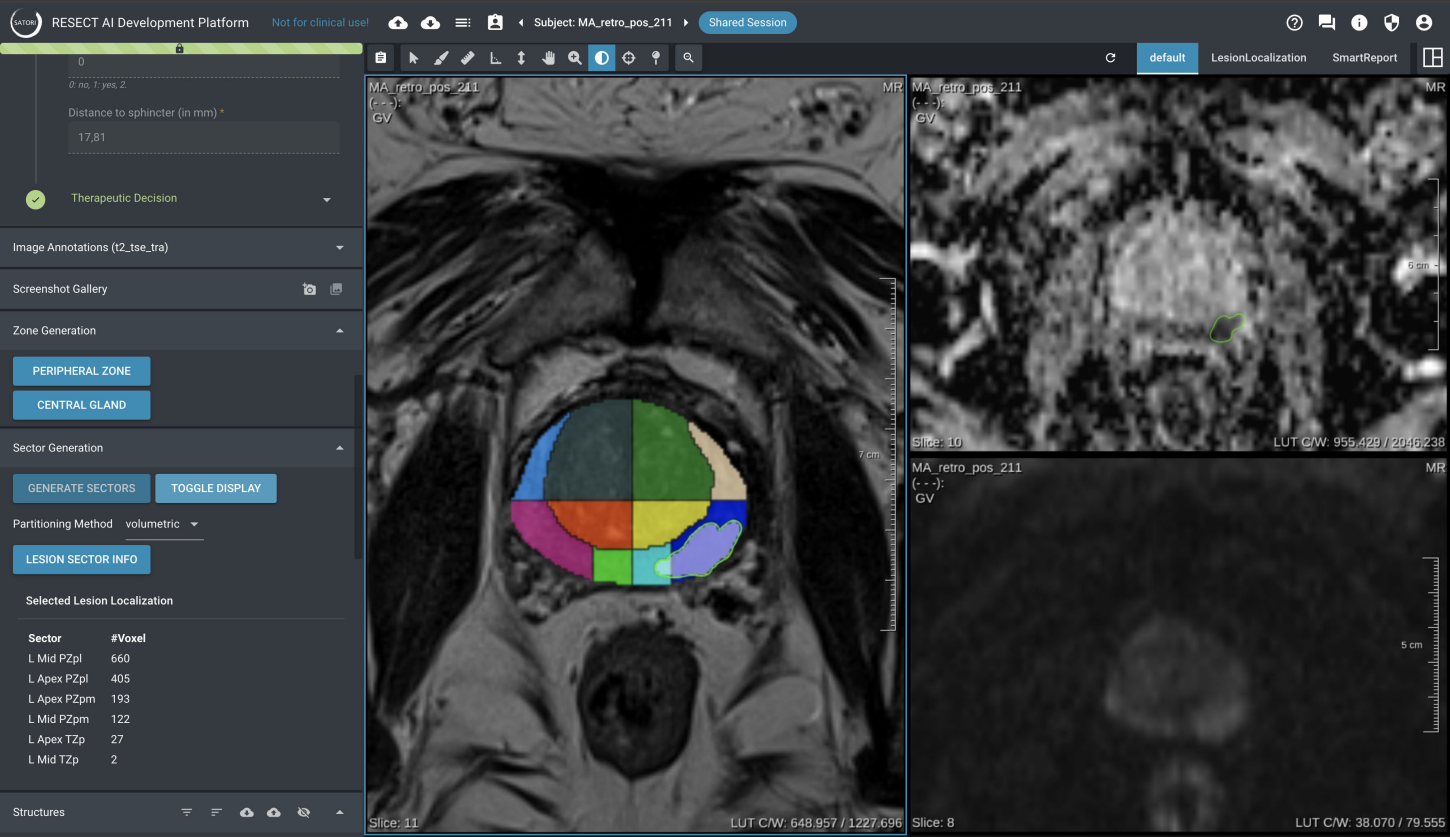
Software prototype of the International Radiomics Platform, a public private partnership of German and Austrian radiological societies with Fraunhofer MEVIS. Conventional parameters such as PSA levels, Gleason Scores, ADC values and therapeutic decisions are displayed in the left-hand column. Images and segmentations are presented in a three-viewer-layout on the right-hand side.

A software prototype based on the International Radiomics Platform (IRP) – initiated by the German and Austrian radiological societies and developed by Fraunhofer MEVIS using its CuraMate toolkit (10) – was implemented as an end-to-end, cloud-based workflow (**Figure 1**). It comprises unified data collection, radiological image interpretation, structured reporting, and quantitative image analysis (for further details see workflow diagram in **Supplementary Figure 1**). Prior to upload, all clinical and imaging data were fully de-identified to comply with data protection regulations. Systematic or targeted prostate biopsy results were imported postoperatively, enabling multimodal feature extraction and model building for three endpoints: extracapsular extension (ECE), positive surgical margins (PSM) after resection, and nerve-sparing approach decision, which was defined as the documented intraoperative or preoperative surgical plan to perform unilateral or bilateral nerve-sparing prostatectomy vs. non-nerve-sparing (wide-excision) prostatectomy, as recorded in the surgical report.

### 2.3 MRI protocol

All patients underwent multi-parametric or bi-parametric MRI of the prostate using a variety of clinical MRI scanners and both 1.5 Tesla (T) and 3.0 T field strengths, ensuring broad heterogeneity and external validity. Minimum imaging requirements included a transverse T2-weighted sequence with a maximum slice thickness of 3 mm and an apparent diffusion coefficient (ADC)-map.

### 2.4 Image postprocessing and radiomics analysis

All image segmentations and image annotations were performed within the IRP. Four investigators defined 3D volumes of interest (VOIs) of tumoral lesions on T2-weighted images and ADC maps, with all delineations reviewed by two board-certified radiologists with more than 5 and 10 years of experience in prostate MRI. The largest lesion or the lesion with lowest ADC-values was selected as the index lesion.

Peripheral zone, transition zone and whole prostate were segmented manually in the first 90 retrospective cases. The segmentations were used to train an nnU-Net to automate prostate zone segmentations (11), which were subsequently reviewed and, if necessary, manually corrected, to ensure anatomical accuracy. Tumor-to-capsule contact length was computed based on the segmentations. Landmarks for neurovascular bundles, urethral sphincter and prostatic apex were marked to compute tumor proximity.

Radiomics features were extracted from both the whole prostate and index lesion using PyRadiomics (default MRI settings, no resampling) (12). The feature set included shape, first-order, and texture features based on GLCM, GLRLM, GLSZM, and GLDM, computed on the original image as well as on LoG-filtered (σ = 1, 3, 5 mm) and wavelet-transformed versions of the images. Overall, this resulted in 1404 radiomics features per patient.

### 2.5 Conventional parameters

For the purposes of this study, ‘conventional parameters’ refers to laboratory, pathological, and clinical variables obtainable in routine clinical care without dedicated quantitative MRI-based image analysis. These include PSA, PSA density, digital rectal examination (DRE) findings, biopsy Gleason score, TURP history, and the IIEF-5 score (see **Supplementary Table 1** for complete data dictionary).

Additionally, 13 imaging-derived parameters were included - PI-RADS scores, MRI T-stage, capsule/apex distances, neurovascular and sphincter involvement and length of capsular contact. For the index lesion, tumor volume, mean ADC, primary localization, ECE grade based on Mehlarivand et al. (13), and radiological assessment of ECE were also recorded. Quantitative imaging parameters such as tumor volume, mean ADC, tumoral distance to capsule or tumor-capsule contact length were automatically computed on the platform. Qualitative features such as ECE grading were determined by the radiologists.

### 2.6 ML-based data analysis and prediction

The prediction models integrated into the IRP prototype were used exclusively for research purposes and did not influence clinical treatment decisions for any patient included in this study. Data analysis and predictive modeling were performed in Python with the scikit-learn package (version 0.24.2) (14). Random Forest classifiers were adopted for their robustness and low hyperparameter sensitivity (15, 16). However, when combining binary or categorical features with continuous features, as done in this study, Extremely Randomized Trees classifiers have been shown to be advantageous (17). While being similar to Random Forests, they use one random split per feature and therefore all features have the same chance of being selected for splitting. For the hyperparameters, the default settings of scikit-learn were used.

To account for the large number of radiomics features and their correlation among each other, a feature selection using Fast Correlation-Based Filter was applied (18). This method determines the number of selected features by itself and does not have any hyperparameters. However, it requires features to be discretized, which was done by standard-normalizing and thresholding at -0.5 and 0.5, yielding three discrete values as recommended for similar methods (19).

The full pipeline consisting of median imputation, discretization, feature selection, training, and validation was performed in a 5-fold cross-validation and repeated 10 times with different random folds to get a reliable estimate of model performance. To investigate the benefit of radiomics, for each endpoint two models were built, one using clinical parameters only and one including radiomics features. Endpoint values were not imputed, i.e. for each endpoint patients with missing information were excluded.

Model discrimination was evaluated by area under the ROC curve (AUC) with 95% confidence intervals (CI), the latter derived by bootstrapping (1,000 samples) (20). Overlap of CIs allowed direct comparison at a 5% significance level.

## 3 Results

### 3.1 Patients and prostate cancer characteristics

The full cohort consisted of 249 patients (mean age: 66.9 years) with mean prostate-specific antigen levels of 10.7 ng/mL and an average of 1.3 suspicious lesions per patient on multiparametric MRI (**Table 1**). 86% of patients had PCa with Gleason scores >= 3 + 4.

**Table 1:** Patient characteristics.

Variables	Retrospective group	Prospective group	Total
Patients (n)	176	73	249
Age (mean ± SD)	66.3 (7.2)	68.2 (7.3)	66.9 (7.3)
PSA (mean ± SD)	11.3 (18.9)	9.8 (9.0)	10.7 (15.9)
Number of suspicious lesions (mean ± SD)	1.3 (0.5)	1.2 (0.5)	1.3 (0.5)
Gleason score at radical prostatectomy (%)			
<= 3 + 3	30 (17)	3 (4)	33 (13)
3 + 4	78 (44)	39 (53)	117 (47)
4 + 3	48 (27)	14 (19)	62 (25)
>= 4 + 4	19 (11)	17 (23)	36 (14)
Pathologic stage (%)			
T2a	15 (9)	11 (15)	26 (10)
T2b	2 (1)	0 (0)	2 (1)
T2c	113 (64)	37 (51)	150 (60)
T3a	18 (10)	17 (23)	35 (14)
T3b	24 (14)	8 (11)	32 (13)
Extracapsular extension (%)	42 (24)	25 (34)	67 (27)
Positive surgical margins (%)	24 (14)	21 (29)	45 (18)
Nerve-sparing approach decision (%)	96 (55)	32 (44)	128 (51)

### 3.2 Software prototype

The cloud-based platform enabled the seamless import of pseudonymized DICOM-images alongside clinical variables (**Figure 1**). The software prototype is further explained in the **Supplementary Material**.

### 3.3 Clinical decision support and outcome prediction

**Figure 2:**
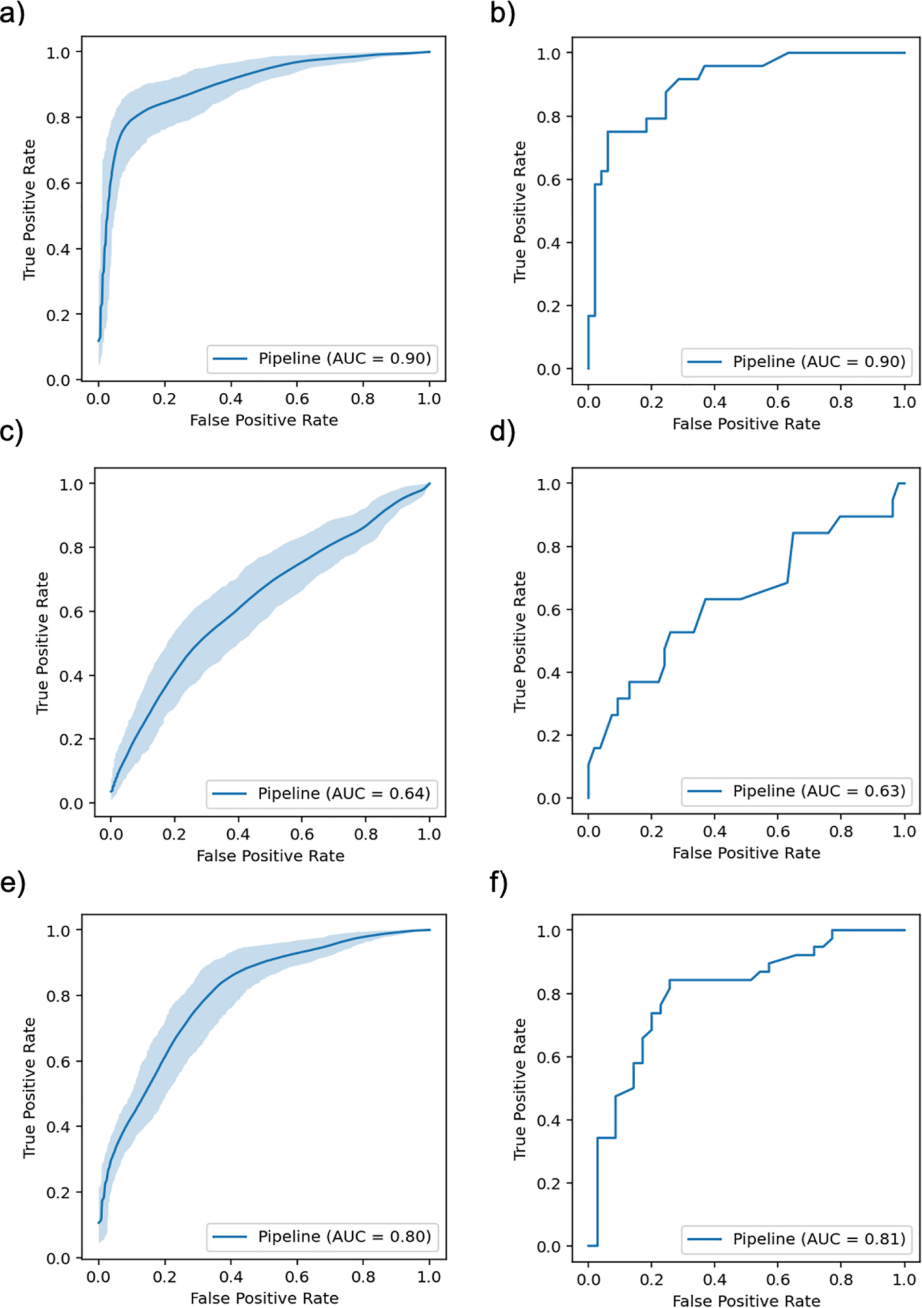
ROC performance of prediction models including conventional parameters, imaging parameters and radiomics parameters: Extracapsular extension **(a, b)**, positive surgical margins **(c, d)**, nerve-sparing approach decision **(e, f)**. Results for 10x5-fold cross validation **(a, c, e)** and training on retrospective and validation on prospective cohort **(b, d, f)**.

The predictive model performance for the three endpoints is summarized in **Table 2**; **Figure 2**.

**Table 2:** Predictive model performances for the three endpoints shown as mean AUCs (with 95% CIs) from 10×5-fold cross-validation and retrospective-to-prospective cohort transfer. The table includes the number of cases and the target distribution per endpoint.

	n	Conventional, imaging and radiomics parameters:	Conventional and imaging parameters only:	Conventional parameters only:
		10x5-fold cross validation	10x5-fold cross validation	10x5-fold cross validation
		Train retrospective – test prospective	Train retrospective – test prospective	Train retrospective – test prospective
**Extracapsular extension**	246 (66 yes, 180 no)	0.90 (0.86, 0.94) 0.90	0.90 (0.86, 0.94) 0.91	0.71 (0.63, 0.77) 0.77
**Positive surgical margins**	248 (45 yes, 203 no)	0.64 (0.57, 0.72) 0.63	0.60 (0.52, 0.68) 0.67	0.50 (0.41, 0.58) 0.49
**Nerve-sparing approach decision**	249 (133 yes, 116 no)	0.80 (0.75, 0.85) 0.81	0.79 (0.73, 0.83) 0.79	0.81 (0.76, 0.86) 0.82

Using conventional parameters for outcome prediction such as PSAD or DRE status without information from MRI, the models achieved AUCs of 0.71 (95% CI: 0.63–0.77) for extracapsular extension, 0.81 (0.76–0.86) for nerve-sparing approach decision, and 0.50 (0.41–0.58) for prediction of positive surgical margins (PSM). Consistent results were observed when algorithms were trained solely on the retrospective cohort and validated on the prospective cohort.

After integrating imaging derived information such as PI-RADS scores or tumor-capsule contact lengths, prediction of ECE could be significantly improved with an AUC of 0.90 (95% CI: 0.86-0.94). Prediction of PSM and nerve-sparing approach decision could not be improved (AUC 0.79, 95% CI: 0.73-0.83; and 0.60, 95% CI: 0.52-0.68).

Notably, models trained on conventional, imaging-derived and radiomics parameters achieved very similar AUCs of 0.90 (CI: 0.86–0.94) for extracapsular extension, 0.80 (CI: 0.75–0.85) for nerve-sparing, and 0.64 (CI: 0.57–0.72) for PSM, which were within confidence intervals of the prediction models that used simple imaging-derived parameters.

## 4 Discussion

This study presents the development and prospective validation of a fully integrated, cloud-based International Radiomics Platform (IRP) for prostate cancer diagnostics, seamlessly unifying image interpretation, structured reporting, and multimodal data analysis for patients with diagnosed or suspected prostate cancer. By leveraging this workflow, we demonstrate that integrated clinical and imaging data management can significantly enhance both the assessment and predictive accuracy of key surgical outcomes, namely extracapsular extension, positive surgical margins (PSM), and potentially nerve-sparing approach decisions, for radical prostatectomy candidates.

Interestingly, the incorporation of simple imaging-derived parameters such as PI-RADS score and tumor-capsule contact length significantly improved the prediction of ECE and PSM. However, quantitative radiomics features did not improve predictive accuracy, compared to well established conventional and imaging-derived parameters. This finding underlines the strength of established markers, the value of imaging, and the necessity for rigorous external validation of novel features. Our platform achieved predictive performance comparable to recent deep-learning approaches (21–23) with the additional benefit of transparent interpretability. Predictive modeling in our cohort achieved maximum AUCs of 0.90 for extracapsular extension, 0.64 for positive surgical margins, and 0.80 for nerve-sparing decision-making, respectively.

Our quantitative image analysis protocol – comprising automated annotation, segmentation, and standardized radiomics extraction – enabled machine learning-based individualized prediction of PSM, ECE, and optimal surgical approach as decision support tools. Historically, ECE and PSM risk prediction has relied predominantly on clinical and basic imaging-derived data (24–26), as embodied in models such as the updated Partin Tables, which use clinical stage, PSA, and biopsy Gleason grade to estimate adverse pathologic findings (24). Recent studies have confirmed that MRI-derived parameters such as capsular contact length, mean ADC values, and anatomical lesion location are independent, high-performing predictors of ECE and PSM (27). A simple prediction model by Park et al. included tumor-capsule contact length, PI-RADs score, and tumor located at the apex and/or posterolaterally, to estimate the risk of PSM (4). Mean ADC values as a simple predictor were similarly accurate in predicting PSM as a multivariate model including ADC values, site and laterality, with AUC values of 68.2% and 70.0%, respectively (28). ADC values are well-known as an independent predictor of ECE (29). Our findings confirm the importance of these traditional imaging biomarkers.

Recent advances in radiomics and machine learning have generated promising results for non-invasive outcome prediction (21, 30–35). For example, He et al. reported that radiomics features from ADC maps alone vs. combined with clinical variables improved AUCs for the prediction of ECE (0.625 vs. 0.728) and PSM (0.733 vs. 0.766), respectively (36). These findings exemplarily demonstrate that radiomics models are often improved by including conventional clinical parameters (37). Conversely, previous studies have demonstrated that the inclusion of imaging parameters yields only marginal improvements in the prediction of PSM, with reported AUCs of 0.74 (38). Our reported maximum AUC of 0.64 is at the lower end of reported values and may better reflect the current consensus that predictability of PSM is limited by complex anatomical variability, variable iatrogenic resection margins and also depends on the surgeon’s experience (39). Direct comparison with established nomograms such as the MSKCC nomogram was not feasible since they were developed for different endpoints (40).

Recent meta-analyses and systematic reviews have highlighted that robust algorithms with prospective validation and reproducible workflows remain scarce, with generalizability across MRI systems and clinical sites still a major hurdle (37, 41). Our findings – derived from both retrospective and prospective patient cohorts encompassing a wide spectrum of MRI protocols – could not demonstrate an additional value of radiomics features. This confirms that identification of robust radiomics parameters with predictive power remains a challenge in real-world settings, and there is a persistent demand for reproducible, interpretable, and generalizable radiomics biomarkers in clinical care.

A distinctive aspect of this study was the prediction of the decision to perform nerve-sparing radical prostatectomy to evaluate the extent to which multimodal data reflects surgical decision-making. However, beyond oncological imperatives such as tumor proximity to neurovascular bundles and extracapsular extension risk, the choice of surgical technique is governed by multifaceted patient-specific factors not captured in our dataset, including baseline erectile function, urinary continence and individual patient preferences. These unmeasured variables likely account for the moderate predictive performance of our models (AUC 0.78-0.81). Notably, the negligible impact of imaging-derived biomarkers on these predictions warrants further investigation, as it suggests a potential misalignment between imaging features and real-world clinical decision making.

Previous studies evaluating AI in prostate cancer imaging have focused primarily on the detection of prostate cancer, classification of suspicious lesions and decision support in biopsy management (42, 43). Several software prototypes and commercially available products have addressed prostate cancer diagnosis and imaging. For instance, Tamposis et al. have implemented a software platform at the university hospital of Larisa in Greece with a focus of data integration and interoperability aiming at developing a risk calculator (44). Regarding prostate segmentations, several DL-based algorithms have been evaluated (45–48) and a number of applications are commercially available and FDA-approved (49, 50). These may be used for automated calculation of volume-standardized PSA levels (PSA density), as well as for MRI-ultrasound-fusion biopsy as well as radiotherapy planning (9).

Technologically, our platform addresses the pivotal requirements for clinical integration: seamless import of pseudonymized multimodal data, vendor-neutral segmentation and annotation, automated calculation of relevant imaging metrics, and direct connectivity to hospital IT environments, including PACS. Such interoperability will help accelerate radiological reporting and supports interdisciplinary communication with urology teams. In addition, the aggregation of annotated imaging and clinical information on the platform will enable future research, federated learning initiatives, and the external validation essential for multi-institutional standards. At the same time, integrated data collection on a cloud-based platform may enable future quantitative image analysis and machine learning research by establishing an annotated data repository (51).

The principal innovation of our approach is the longitudinal, end-to-end support of the diagnostic and treatment planning process. Radiology thus stands poised to take a central role in integrated prostate cancer diagnostics by providing a robust, explainable, and interoperable foundation for multimodal data analytics in therapy planning with a central role of diagnostic imaging along the patient pathway.

Several limitations of this study should be acknowledged. The prostate cancer lesions on MRI were not directly mapped to whole-mount specimens so that there might be uncertainty as to the extension and location of prostate cancer lesions. The study included imaging data from several different MRI scanners and manufacturers, and imaging protocols have not been standardized, which adds to the heterogeneity of imaging data. While this likely compromised quantitative image analysis, it represented a generalizable approach representative of routine clinical care.

A key limitation of our single center study is that all included patients were pre-selected for radical prostatectomy based on clinical decisions made prior to modeling. Consequently, no alternative treatment pathways (such as radiotherapy or active surveillance) were represented, limiting the generalizability of outcome prediction to the broader prostate cancer population. All models should be regarded as specific to surgical cohorts, and do not yet inform choice between treatment modalities or outcome probabilities for diverse clinical scenarios. Due to ethical and regulatory constraints, the experimental algorithm could not be applied in real-time clinical decision-making, since an influence on treatment choices needed to be precluded. Moreover, several endpoints demonstrated only moderate predictive performance, consistent with the known complexity of multifactorial oncological outcomes. In addition, the retained predictive performance of conventional parameters alone for nerve-sparing approach decisions (AUC 0.81) - equivalent to models incorporating imaging data - suggests that established clinical indicators already capture most of the variance currently measurable in our dataset. These results must be interpreted cautiously: the nerve-sparing decision integrates patient-reported outcomes, baseline erectile and urinary function, and intraoperative anatomical assessment, none of which were systematically captured in our model. Our findings should therefore not be interpreted as evidence that imaging or radiomics are uninformative for nerve-sparing planning, but rather that the current feature set does not yet capture the full complexity of this clinical decision. As an exploratory proof-of-concept study, these findings should not be interpreted as establishing a clinical decision support. External, multi-center validation in larger and more diverse cohorts including decision curve analysis and calibration curves is required before clinical deployment.

In conclusion, in the present exploratory study, we demonstrated the clinical feasibility and translational potential of a platform-based, interdisciplinary analysis framework in supporting outcome prediction and personalized decision-making for prostate cancer surgical planning. Full workflow integration accelerates multimodal data analysis, streamlines radiological reporting, and lays the groundwork for next-generation precision oncology in routine clinical practice. Our results establish a proof-of-concept for comprehensive, prospective data integration platforms and underline the complexity and limited clinical utility of radiomics-derived biomarkers in clinical routine.

## Data Availability

The datasets presented in this article are not readily available since they contain protected health information. Requests to access the datasets should be directed to the corresponding author.
