# Supplementary material for: From AI-based image analysis to surgical decision support in prostate cancer: interdisciplinary application of the international radiomics platform
Source: Front Oncol. 2026 May 29;16:1816463. doi: 10.3389/fonc.2026.1816463 (PMC13259821; doi:10.3389/fonc.2026.1816463)
Supplement: Supplementary file 1 [file DataSheet1.pdf]

Supplementary Material

Contents:

Conventional and imaging-derived parameters - data dictionary …………….………2
International radiomics platform (IRP) - workflow diagram …………………………….4

Table S1: Data dictionary: conventional and imaging-derived parameters

**Variable**	**Explanation**	**units**
PSA	Prostate-specific antigen level	ng/ml
PSA-Density	Prostate-specific antigen level / prostate volume derived from DL-based volumetry	ng/ml/ml
DRE	Digital rectal examination with suspicious finding	dichotomous
Biopsy Gleason Score	Gleason Score derived from systematic biopsy and targeted biopsy (when available)	Gleason Scores
TURP history	Transurethral resection of the prostate	dichotomous
IIEF-5 Score	International index of erectile function	Scores from 5 to 25
PI-RADS Score	Protate Imaging-Reporting and Data System	Scores from 1 to 5
MRI T-stage	Tumor stage according to 8th edition of TNM	T categories from T2 to T4
Tumor-capsule distance	Smallest distance between the tumor boundary and the capsule on transversal T2 images	mm
Tumor-apex distance	Smallest distance between the tumor boundary and the apex of the prostate on transversal T2 images	mm
Neurovascular bundle involvement, left and right	Infiltration of neurovascular bundles determined by the radiologist	dichotomous
Sphincter involvement	Infiltration of the sphincter determined by the radiologist	dichotomous
Tumor-capsule contact length	Distance of contact between the tumor and the capsule on transversal T2 images	mm

Volume	Volume of the tumor segmentation on transversal T2 images	ml
Mean ADC	Mean ADC (Apparent Diffusion Coefficient) values based on tumor segmentation on ADC-maps	ml
Localization	Sectors the tumor occupied on the ACR Sector Map based on PI-RADS version 2.1	ACR Sector Map
EPE grade	Extraprostatic extension grade based on Mehlarivand et al.	Scale from 1 to 3
ECE	Extracapsular extension determined by the radiologist	dichotomous

Figure S1: International Radiomics Platform (IRP) - Workflow diagram

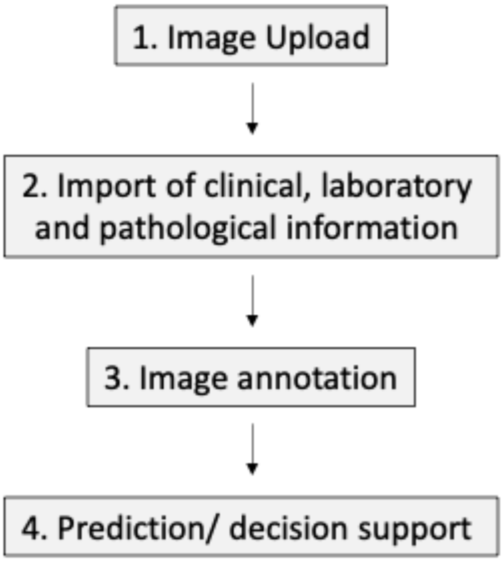

1. **Image Upload:** Prostate MRI DICOM images were pseudonymized on site, and then uploaded to the cloud-based IRP via its web-interface.
2. **Import of further information:** Clinical, laboratory, and pathological information could either be entered manually or uploaded as a csv-file that contained the information described in Material and Methods and in the data dictionary.
3. **Image annotation:** The images were segmented by a deep learning-based algorithm, that created segmentations of the peripheral zone, transition zone, and whole prostate. The segmentations were reviewed and corrected, when necessary. Tumors were segmented on T2-weighted images and ADC-maps manually. Markers for the neurovascular bundles, prostate apex and sphincter were defined on T2-weighted images.
4. **Prediction and decision support:** The prediction models were first developed offline in Python. When the training was complete, the algorithms were integrated into the platform, so that data sets could be prospectively analyzed online.
